# Preparative leukapheresis impairs autologous donor hemostasis and platelet function

**DOI:** 10.1016/j.rpth.2026.106819

**Published:** 2026-07-13

**Authors:** Veronika Dill, Philipp Blüm, Laura-Vanessa Stegmaier-Diaz, Georg Mößmer, Markus Thaler, Patrick Möhnle, Florian Bassermann, Martin Hildebrandt

**Affiliations:** 1 Department of Internal Medicine III Haematology and Oncology, Technical University of Munich (TUM) School of Medicine and Health, TUM University Hospital, Munich, Germany; 2 German Cancer Consortium (DKTK), partner site Munich, Technical University of Munich, Munich, Germany; 3 Institute of Transfusion Medicine and Immunology, Medical Faculty Mannheim, Heidelberg University, German Red Cross Blood Service Baden-Württemberg-Hessen, Mannheim, Germany; 4 Technical University of Munich (TUM) School of Medicine and Health, Institute of Clinical Chemistry and Pathobiochemistry, TUM University Hospital, Munich, Germany; 5 Division of Transfusion Medicine, Cell Therapeutics and Hemostaseology, University Hospital, Ludwig Maximilian University, Munich, Germany; 6 Center for Translational Cancer Research (TranslaTUM), TUM School of Medicine, Technical University of Munich, Munich, Germany; 7 Bavarian Cancer Research Center (BZKF), Erlangen, Germany

**Keywords:** blood coagulation factors, blood platelets, hemostasis, leukapheresis

## Abstract

**Background:**

Preparative apheresis is performed to produce blood or stem cell products. A rare but serious complication is bleeding due to the inherent loss of platelets and coagulation factors.

**Objectives:**

The aim of this study was to provide a comprehensive analysis of hemostasis in autologous donors undergoing preparative apheresis and to assess bleeding complications in this cohort.

**Methods:**

In this prospective observational study, a large panel of cellular components and coagulation factors was quantified both immediately before and after apheresis of hematopoietic stem cells (*n* = 50) and lymphocytes (*n* = 29) for autologous cellular therapies.

**Results:**

Postapheresis, we observed a small decrease (5.9%) in prothrombin time, while international normalized ratio, partial thromboplastin time, and thrombin time remained unaffected. Single component analysis revealed a median loss of factor (F)XIII of 15%, fibrinogen of 17.9%, antithrombin of 13.9%, von Willebrand factor (VWF) antigen of 18.7% and VWF activity of 18.2%. In terms of cellular components, we found a median reduction of 25.6% in platelet count, 19.4% reduction in leukocyte count, and 7.1% in hemoglobin level. Platelet aggregation after stimulation with arachidonic acid, adenosine diphosphate, and thrombin-activated peptide-6 was reduced. One patient (1.3%) with evident FXIII deficiency developed minor bleeding and required surgical intervention.

**Conclusion:**

Our analysis shows that hemostasis and platelet function are compromised after apheresis in autologous donors, despite mostly unaltered global coagulation parameters. These findings may be of value in the clinical management of apheresis, particularly regarding procedure-related bleeding events.

## Essentials

• Bleeding is a rare but potentially serious complication of preparative apheresis.
• Hemostasis was evaluated immediately before and after cell apheresis in 79 autologous donors.
• FXIII, fibrinogen, antithrombin, VWF, and platelet aggregation were reduced after apheresis.
• The apheresis-related impairment of hemostasis may require more attention.

## Graphical abstract

**Figure:**
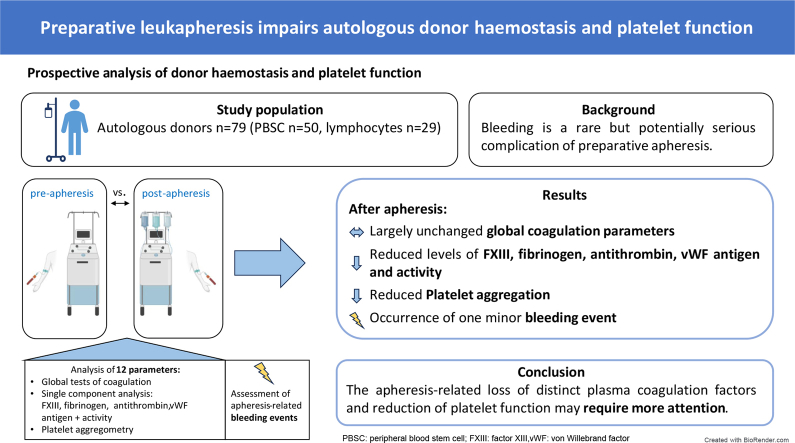

## 1 Introduction

Preparative apheresis, ie, the collection of blood components with a cell separator, is widely used to obtain blood or stem cell products. Albeit a standardized procedure, it carries some risk of complications [1] such as bleeding events, among others. Several factors may contribute to the risk of bleeding and should be considered as they affect both autologous and healthy allogeneic donors. Primarily, a loss of cellular components is inherent to the process. Previous studies have shown a significant decrease in platelet counts in donors after allogeneic and autologous stem cell collection [2,3]. A platelet loss of 28% has been reported in healthy donors [3]. In general, a platelet decreases of 20% to 30% can be expected, depending on the preapheresis level [4]. This is also taken into account in national guidelines regarding donor clearance and exclusion criteria [5].

Limited information is available on the effect of preparative apheresis on the loss of plasma components. Platelet apheresis is known to affect prothrombin time (PT) and plasminogen levels in healthy donors [6]. While certain exclusion criteria for plasma donation have been established, for instance, in the German Medical Association’s guidelines for hemotherapy [7], there are no corresponding recommendations for cell apheresis. However, a considerable amount of plasma is removed during this procedure. Given that patients often develop severe thrombocytopenia after stem cell mobilization chemotherapy, the plasma loss may affect the concentration and activity of coagulation factors and further increase the risk of bleeding [[8], [9], [10]]. Furthermore, the use of acid citrate dextrose formula-A (ACD-A) anticoagulant during cell apheresis may also affect hemostasis.

Plasma protransglutaminase factor (F)XIII has not yet been evaluated in the context of cell apheresis. When designing this study, we were particularly interested in plasma FXIII. Activated by thrombin, FXIII catalyzes the cross-linking of fibrin to form a stabilized fibrin network, thereby protecting the fibrin clot from degradation [11]. Clinically, FXIII deficiency is associated with severe and potentially life-threatening bleeding, particularly in soft tissue [12]. While inherited forms of FXIII deficiency are rare, acquired deficiencies—in which FXIII levels drop to 20% to 70% of normal due to decreased synthesis or increased consumption—have been described in cases of liver and autoimmune diseases, as well as in malignant disorders [[12], [13], [14], [15], [16]].

As patients undergoing preparative apheresis may be at a higher risk for acquired disorders of primary and secondary hemostasis, and as FXIII is not tested routinely as part of the global coagulation assays [17], it seemed reasonable and clinically relevant to implement an extended coagulation monitoring including FXIII activity. The aim of this study was to evaluate the effects of preparative apheresis on hemostasis including FXIII and platelet function in patients recruited for autologous stem cell collection as well as to assess the occurrence of bleeding complications and a possible association with detected deficiencies.

## 2 Patients and Methods

### 2.1 Design

This single-center, prospective study enrolled 79 autologous donors who underwent sequential preparative apheresis for hematopoietic stem cell cells (*n* = 50) or lymphocytes intended for advanced therapy medicinal products (*n* = 29) at our institution between January 2023 and April 2024. Informed written consent was obtained from each patient. The study was approved by the local ethics committee (ethics approval 2022-441-S-SR). To address any selection bias, consecutive recruitment of autologous donors was performed.

### 2.2 Leukapheresis

Apheresis procedures were performed with the TERUMO Blood and Cell Technologies Spectra Optia Apheresis System using the kMNZ 11.3 mononuclear cell program (Terumo BCT). ACD-A was used as an anticoagulant during apheresis at a whole blood:ACD-A ratio of 1:12 and reduced to 1:14 after 1 hour or in the case of a citrate reaction, in accordance with the manufacturer’s recommendations. If the donors presented with normal platelet counts, a 1:12 ratio was preferred to prevent clotting.

The end fluid balance (EFB) was calculated by the difference between the fluids added (ACD-A and rinseback) and the fluids taken (collected volume, drained saline solution, and tubing set).

Calcium was only administered to treat symptoms of hypocalcemia. A total of 47 donors required calcium substitution. Of these, 23 received 2.25 mmol calcium gluconate intravenously and 4 received 4.5 mmol. Eleven donors received 500 mg effervescent tablets, 3 received 1000 mg, and 2 received 1500 mg; 2 were given a combination of 2.25 mmol and 500 mg, and 2 others received 4.5 mmol and 500 mg. If indicated, systemic anticoagulation was discontinued at least 48 hours prior to the procedure.

### 2.3 Sample collection

Citrate, hirudin, and EDTA anticoagulated samples were collected from each donor both before and immediately after apheresis. The postapheresis sample was taken after rinseback from the lines after flushing. Samples were analyzed immediately after collection at the Institute of Clinical Chemistry and Pathobiochemistry of our University Hospital.

For coagulation factor analysis, citrate anticoagulated samples were centrifuged at 2300 *g* for 9 minutes at room temperature.

### 2.4 Laboratory investigations

Samples were analyzed for PT, activated partial thromboplastin time (PTT) and von Willebrand factor (VWF) activity using the Thromborel S, Pathromtin siliconized latex, and Innovance VWF Ac reagents, respectively, on a Behring Coagulation System (BCS XP System) (all from Siemens Healthcare Diagnostics). Fibrinogen was calculated as derived fibrinogen from the PT reaction curve. VWF antigen, FXIII, and antithrombin were quantified as well on the BCS XP system employing the Liaphen VWF antigen, Biophen FXIII, and Biophen antithrombin Liquid Reagent Technology (anti-human-Xa) reagents, respectively (all from Hyphen Biomed). Thrombin time (TT) was measured by use of the Test Thrombin reagent (Siemens Healthcare Diagnostics) on a KC4A coagulometer (ABW Medizin und Technik GmbH).

Whole blood impedance aggregometry was performed on a Multiplate analyzer (Roche). Samples were stimulated with arachidonic acid, adenosine diphosphate (ADP), and thrombin-activated peptide-6 (TRAP) according to the manufacturer’s instructions, with results expressed as area under the curve. Complete blood count (leukocyte count, platelet count, and hemoglobin) was performed on a Sysmex XN-2000 or XN-9100 analyzer (Sysmex). All measurements have been established by the Institute of Clinical Chemistry and Pathobiochemistry and are carried out regularly as part of routine diagnostics, in accordance with the respective protocol.

### 2.5 Outcome analyses

The primary outcome was to evaluate the reduction in FXIII activity attributable to the apheresis procedure. Assuming a hypothetical loss of FXIII activity of 20%, with an *R*^2^ value of 0.1, a statistical power of 0.8 and an alpha level of 0.05, the calculated sample size was *n* = 73. Secondary analyses were designed to quantify changes in the other coagulation factors as described above and to report the incidence of bleeding complications. For bleeding complications, the bleeding event and the clinical and laboratory outcomes were assessed and documented. Additionally, a post-hoc analysis of calcium levels before and after apheresis was performed.

### 2.6 Statistical analysis

Paired *t*-tests were used to compare pre- and postapheresis measurements. Patients with missing parameters either pre- or postapheresis were excluded from the respective analysis. Subgroup comparisons between peripheral blood stem cell (PBSC) and lymphocyte collections were performed using either the Mann–Whitney test or Welch’s *t*-test. For correlation analyses, the strength of the association between FXIII loss and apheresis parameters was calculated by Spearman rank correlation, and the functional relationship was described by linear regression analysis.

All reported *P-*values are 2-tailed with a significance level of .05 and were not adjusted for multiple testing. Statistical analyses were performed using GraphPad Prism version 8 (GraphPad) and SPSS version 29.0.2.0 (SPSS Inc).

## 3 Results

### 3.1 Demographic data

Seventy-nine consecutive patients undergoing autologous apheresis at our institution were recruited to participate in the study. Apheresis was performed to collect PBSCs (*n* = 50) or lymphocytes for further processing (*n* = 29) (Table 1). The median age of the donors was 59 years (PBSC 50 years, lymphocytes 60 years, *P* = .34). Patients were diagnosed with plasma cell disorders (*n* = 45), lymphoma (*n* = 30), adult acute lymphoblastic leukemia (*n* = 2), and Ewing’s sarcoma (*n* = 2). The median total blood volume (TBV) of the donors was 5021 mL (PBSCs 5129 mL, lymphocytes 4657 mL; *P* = .08). The median total volume collected including plasma was 493 mL (PBSCs 581 mL, lymphocytes 342 mL; *P* < .0001), representing 9.7% (PBSCs 11.4%, lymphocytes 7.2%; *P* < .0001) of the TBV. The median processed volume was 12,136 mL (PBSCs 11,999 mL, lymphocytes 12,351 mL; *P* = .57). The median consumption of ACD-A was 984 mL (PBSCs 952 mL, lymphocytes 1052 mL; *P* = .04), accounting for 19.96% of the TBV (PBSCs 19.44%, lymphocytes 21.61%; *P* =.001. The EFB was 578 mL (PBSCs 491.5 mL, lymphocytes 847 mL; *P* < .0001), corresponding to 12.44% of the TBV (PBSCs 9.83%, lymphocytes 17.62%; *P* < .0001). The median duration of apheresis was 242 minutes (PBSCs 253 minutes, lymphocytes 240 minutes; *P* = .30; Table 1).

**Table 1:** Patient characteristics and procedural data.

Characteristic		*n*	%	
**Sex**	Female	28	35.4	
	Male	51	64.6	
**Apheresis type**	PBSC	50	63.3	
	Lymphocytes	29	36.7	
**Diagnosis**	Plasma cell disorder	45	57	
	Lymphoma	30	38	
	Acute lymphoblastic leukemia	2	2.5	
	Ewing’s sarcoma	2	2.5	
		**Median**	**IQR (Q1; Q3)**	***P* value**
**Age**	**Total**	**59**	**52; 66**	.34
	PBSC	59	53; 63	
	Lymphocytes	60	52; 71	
**Total collected volume, mL**	**Total**	**493**	**348; 601**	<.0001
	PBSC	581	495; 640	
	Lymphocytes	342	314; 366	
**TBV, mL**	**Total**	**5021**	**4309; 5563**	.08
	PBSC	5129	4432; 5666	
	Lymphocytes	4657	3862; 5438	
**Collected volume/TBV, %**	**Total**	**9.7**	**7.3; 12.3**	<.0001
	PBSC	11.4	9.5; 12.9	
	Lymphocytes	7.2	6.4; 8.9	
**Processed volume, mL**	**Total**	**12,136**	**9812; 13,404**	.57
	PBSC	11,999	9439; 13,411	
	Lymphocytes	12,351	10,888; 13,316	
**Processed volume/TBV**	**Total**	**2.4**	**2.1; 2.7**	.05
	PBSC	2.4	2.0; 2.6	
	Lymphocytes	2.5	2.2; 3.1	
**Infused ACD-A, mL**	**Total**	**984**	**842; 1091**	.04
	PBSC	952	730; 1058	
	Lymphocytes	1052	945; 1150	
**Infused ACD-A/TBV, %**	**Total**	**19.96**	**16.62; 22.62**	.001
	PBSC	19.44	15.04; 21.90	
	Lymphocytes	21.61	19.66; 24.58	
**EFB, mL**	**Total**	**578**	**394; 835**	<.0001
	PBSC	491.5	361.5; 594.5	
	Lymphocytes	847.0	717; 936	
**EFB/TBV (%)**	**Total**	**12.44**	**8.34; 16.99**	<.0001
	PBSC	9.83	6.89; 12.58	
	Lymphocytes	17.62	15.44; 19.95	
**Apheresis time, min**	**Total**	**242**	**210; 277**	.30
	PBSC	253	199; 282	
	Lymphocytes	240	219; 258	

Statistical analysis was performed using the Mann–Whitney test. Plasma cell disorders comprise patients with multiple myeloma, light chain amyloidosis, plasma cell leukemia, and monoclonal gammopathy of renal significance. Lymphoma includes patients with non-Hodgkin’s lymphoma, such as diffuse large B-cell lymphoma, primary mediastinal large B-cell lymphoma, central nervous system lymphoma, T-cell lymphoma and mantle cell lymphoma, and Hodgkin’s lymphoma.

ACD-A, acid citrate dextrose formula-A; EFB, end fluid balance; PBSC, peripheral blood stem cell; TBV, total blood volume.

### 3.2 Donor hemostasis

**Figure 1:**
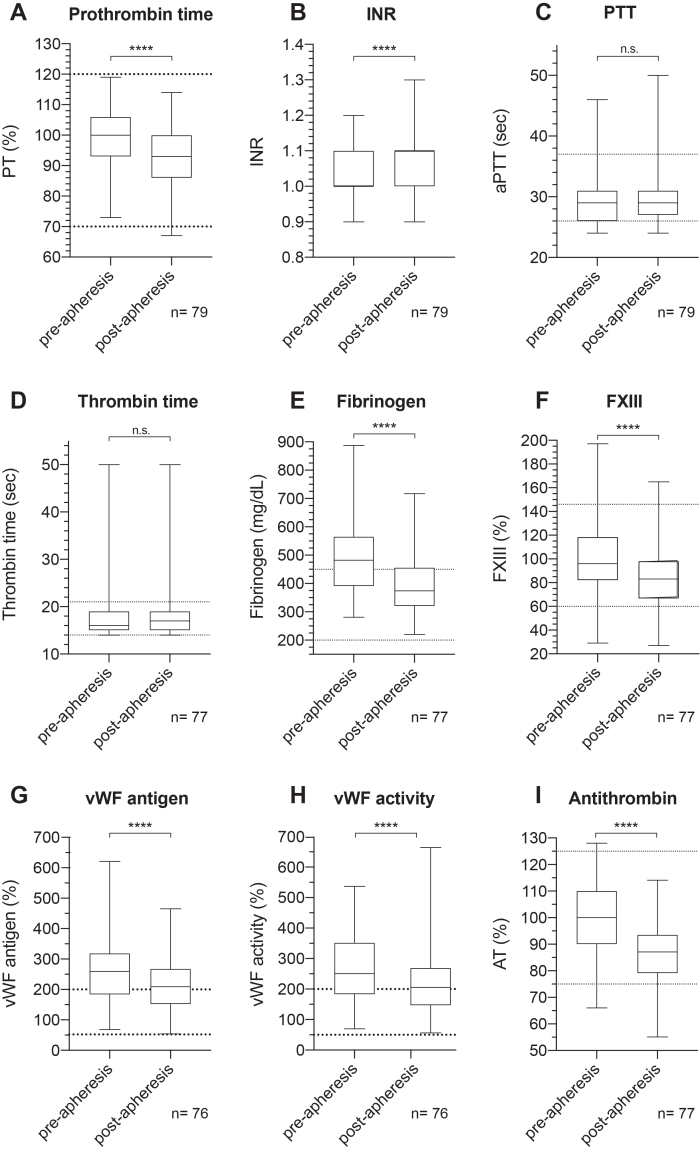
Changes in coagulation factors after preparative apheresis. All panels: Box plots show the median and IQR (25th and 75th). Whiskers indicate minimum and maximum values analyzed. The respective reference ranges of the Institute of Clinical Chemistry and Pathobiochemistry are shown as dotted lines. Only patients with both pre- and postapheresis values available were included. The number of samples analyzed is shown in the figure. Pre- and postapheresis values were analyzed by paired *t*-test. Level of significance .05, ∗∗∗∗*P* < .0001. n.s., not significant. (A) Changes in prothrombin time from pre- to postapheresis (*P* <.0001). (B) Changes in international normalized ratio (INR) from pre- to postapheresis (*P* < .0001). (C) Changes in activated partial thromboplastin time (aPTT) from pre- to postapheresis (*P* = .4048). (D) Changes in thrombin time from pre- to postapheresis (*P* = .3279). (E) Changes in fibrinogen from pre- to postapheresis (*P* < .0001). (F) Changes in coagulation factor XIII (FXIII) from pre- to postapheresis (*P* < .0001). (G) Changes in von Willebrand factor (VWF) antigen from pre- to postapheresis (*P* < .0001). (H) Changes in VWF activity from pre- to postapheresis (*P* < .0001). (I) Changes in antithrombin from pre- to postapheresis (*P* < .0001).

To assess the effect of the apheresis procedure on hemostasis, a panel of coagulation factors was evaluated before and immediately after the apheresis procedure as described above. First, standard parameters of the extrinsic and common coagulation pathways were analyzed. A decrease in PT was observed from median preapheresis levels of 100% to postapheresis levels of 93% (*P* < .0001; Figure 1A), with a median percentage loss of 5.9% (Table 2). The median preapheresis international normalized ratio (INR) increased slightly, from 1.000 to a median postapheresis INR of 1.100 (*P* < .0001) (Figure 1B; Table 2). No statistically significant differences or percentage changes were observed in the intrinsic coagulation pathway as analyzed by PTT (median PTT preapheresis 29 seconds, median PTT postapheresis 29 seconds; *P* = .40) (Figure 1C, Table 2).

**Table 2:** Percentage change in analyzed parameters after apheresis.

Change postapheresis		*n*	Percentage change (median)	Percentage change IQR (Q1; Q3)
**Coagulation factors**	PT	79	−5.9	−9.5; −2.1
	INR	79	0	0; 10
	PTT	79	0	0; 3.7
	TT	76	0	0; 6.7
	Fibrinogen	77	−17.9	−23.35; −13.8
	FXIII	77	−15	−22; −10.5
	VWF antigen	76	−18.65	−23.68; −13.33
	VWF activity	76	−18.2	−23.73; −14.23
	Antithrombin	77	−13.9	−16.7; −10.3
**Blood count**	Platelets	77	−25.6	−36.15; −17.10
	Hemoglobin	79	−7.1	−10.2; −4.4
	Leukocytes	29	−19.4	−30.5; −9.55
**Platelet aggregation**	Arachidonic acid	72	−15.55	−32.08; 5.175
	ADP	73	−23.3	−40.55; −.55
	TRAP	73	−20.1	−36.35; 3.25

**Figure 2:**
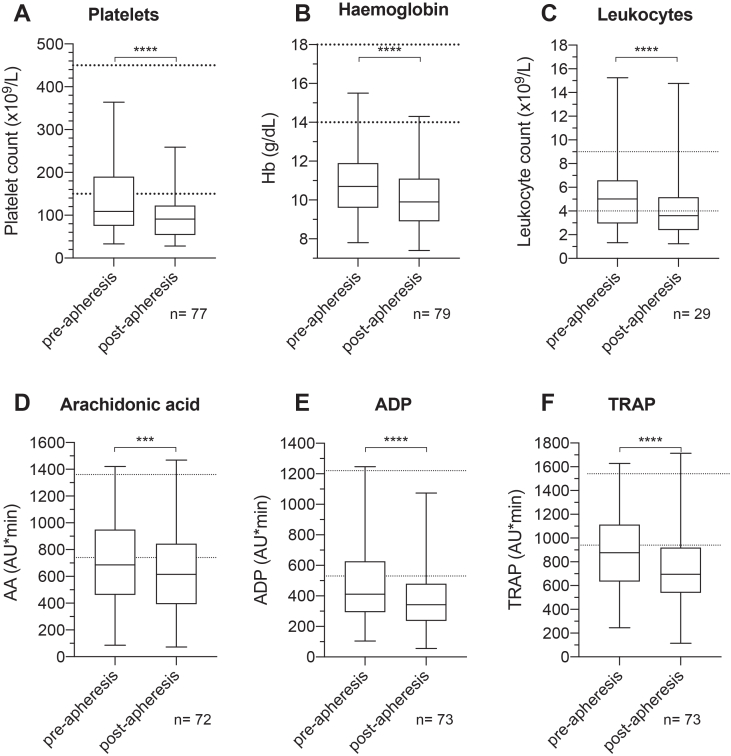
Changes in blood count and platelet aggregation after apheresis. All panels: Box plots show the median and interquartile range (25th and 75th). Whiskers indicate minimum and maximum values analyzed. The respective reference ranges of the Institute of Clinical Chemistry and Pathobiochemistry are shown as dotted lines. Only patients with both pre- and postapheresis values available were included. The number of samples analyzed is shown in the figure. Pre- and postapheresis values were analyzed by paired *t*-test. Level of significance .05, ∗∗∗*P* < .001, ∗∗∗∗*P* < .0001. (A) Changes in platelet count from pre- to postapheresis (*P* < .0001). (B) Changes in hemoglobin from pre- to postapheresis (*P* < .0001). (C) Changes in leukocyte count from pre- to postapheresis (*P* < .0001). Due to the mobilization kinetics in peripheral blood stem cell apheresis patients, only patients undergoing lymphocyte apheresis were included in this analysis. Panels D–F: Changes in platelet aggregation as determined by the area under the curve (arbitrary units × min) of impedance aggregometry (Multiplate) after stimulation with (D) arachidonic acid (*P* = .0007), (E) adenosine diphosphate (ADP) (*P* < .0001), and (F) thrombin-activated peptide-6 (TRAP) (*P* < .0001) from pre- to postapheresis.

Summary of changes in coagulation factors, platelet aggregation, and blood count after preparative apheresis, shown as median percentage of post-apheresis values relative to pre-apheresis values from Figure 1 and Figure 2.

ADP, adenosine diphosphate; INR, international normalized ratio; FXIII, factor XIII; PT, prothrombin time; PTT, partial thromboplastin time; TRAP, thrombin-activated peptide-6; TT, thrombin time; VWF, von Willebrand factor.

Notably, TT remained unaffected by the apheresis procedure, with median values of 16 seconds preapheresis and 17 seconds postapheresis (*P* = .33; Figure 1D), resulting in no percentage change (Table 2). We also observed a reduction in fibrinogen with a median preapheresis concentration of 482 mg/dL and a median postapheresis concentration of 374 mg/dL (*P* < .0001; Figure 1E), indicating a median reduction of 17.9% (Table 2). The median preapheresis FXIII activity of 96% was reduced to 83% postapheresis (*P* < .0001; Figure 1F), representing a median reduction of 15% (Table 2).

To further evaluate the effect of cell apheresis on hemostasis, VWF antigen and VWF activity were analyzed. The median preapheresis VWF antigen of 259% decreased to 209% postapheresis (*P* < .0001; Figure 1G), reflecting a median reduction of 18.65% (Table 2). A similar reduction was observed in VWF activity, with a median preapheresis activity of 250.5% and a median postapheresis activity of 205.5% (*P* < .0001; Figure 1H), resulting in a median reduction of 18.2% (Table 2). Finally, the median preapheresis activity of antithrombin, which has an endogenous anticoagulant function, was 100% before apheresis and decreased to 87% after apheresis (*P* < .0001; Figure 1I), with a median reduction of 13.9% (Table 2).

### 3.3 Donor blood count

As a loss of cellular components, especially platelets, is inherent in cell apheresis [2], platelet count, leukocyte count, and hemoglobin levels were evaluated in our study. To address the changes associated with stem cell mobilization treatment by mobilization chemotherapy and/or granulocyte colony-stimulating factor (G-CSF), the cellular components were analyzed separately for patients undergoing PBSC apheresis after mobilization therapy and patients undergoing lymphocyte apheresis. For leukocyte counts, only patients undergoing lymphocyte apheresis were evaluated, while those undergoing PBSC apheresis were not analyzed due to the variable mobilization kinetics caused by the use of G-CSF.

The median preapheresis platelet count of 109 × 10^9^/L was reduced to 91 × 10^9^/L postapheresis (*P* < .0001; Figure 2A), with a median reduction of 25.6% (Table 2). The median hemoglobin level decreased from 10.7 g/dL before apheresis to 9.9 g/dL after apheresis (*P* < .0001; Figure 2B), in line with a median decrease of 7.1% (Table 2). For lymphocyte donors, the median preapheresis leukocyte count (5.01 × 10^9^/L) was reduced to 3.6 × 10^9^/L postapheresis (*P* < .0001; Figure 2C), representing a median reduction of 19.4% (Table 2).

The PBSC cohort had a lower median platelet count before apheresis (88 × 10^9^/L) compared with the lymphocyte cohort (172 × 10^9^/L, *P* = .0004; Supplementary Figure 1A). This was also observed after apheresis, with median platelet counts of 69 × 10^9^/L in the PBSC group and of 120 × 10^9^/L in the lymphocyte group (*P* = .004). In both cohorts, the platelet counts showed a decrease after apheresis (*P* < .0001; Supplementary Figure 1A). As demonstrated for the entire cohort, postapheresis hemoglobin levels were observed to be lower in the PBSC and lymphocyte apheresis groups (*P* < .0001; Supplementary Figure 1B). The median hemoglobin levels of the subgroups were comparable before apheresis (PBSCs 10.9 g/dL, lymphocytes 10.7 g/dL; *P* = .94) and after apheresis (PBSCs 10.1 g/dL, lymphocytes 9.5 g/dL; *P* = .39).

### 3.4 Donor platelet function

To provide a functional analysis of platelet function, platelet aggregation was assessed by analyzing the area under the curve of impedance aggregometry (Multiplate assay). Upon stimulation with arachidonic acid, we observed a reduction in platelet function with a median preapheresis aggregation response of 685.5 arbitrary units (AU) × min to a median postapheresis aggregation response of 615.5 (*P* = .0007; Figure 2D), yielding a median reduction of 15.55% (Table 2). Similar results were seen upon stimulation with ADP, with a median preapheresis aggregation response of 411 AU × min followed by a median postapheresis aggregation response of 342 AU × min (*P* < .0001; Figure 2E) and a median reduction of 23.3% (Table 2). After stimulation with TRAP, the median aggregation response observed before apheresis decreased from 876 AU × min to 695 AU × min (*P* < .0001; Figure 2F), resulting in a median reduction of 20.1% (Table 2).

Given that platelet aggregometry analyzed by the Multiplate assay is dependent on the platelet count [18], platelet aggregation was evaluated separately in patients with platelet counts >100 × 10^9^/L both before and after apheresis. Again, we observed a significant decrease in platelet function after stimulation with arachidonic acid (*P* = .0003; Supplementary Figure 2A), ADP (*P* < .0001; Supplementary Figure 2B), and TRAP (*P* = .0014; Supplementary Figure 2C).

### 3.5 Calcium levels

An additional post-hoc analysis of calcium before and after apheresis revealed a decrease in median serum calcium levels from 2.28 mmol/L to 2.20 mmol/L after the procedure (*P* < .0001; Supplementary Figure 4).

### 3.6 Decrease in FXIII activity correlates with TBV-related processed volume and collected volume

**Figure 3:**
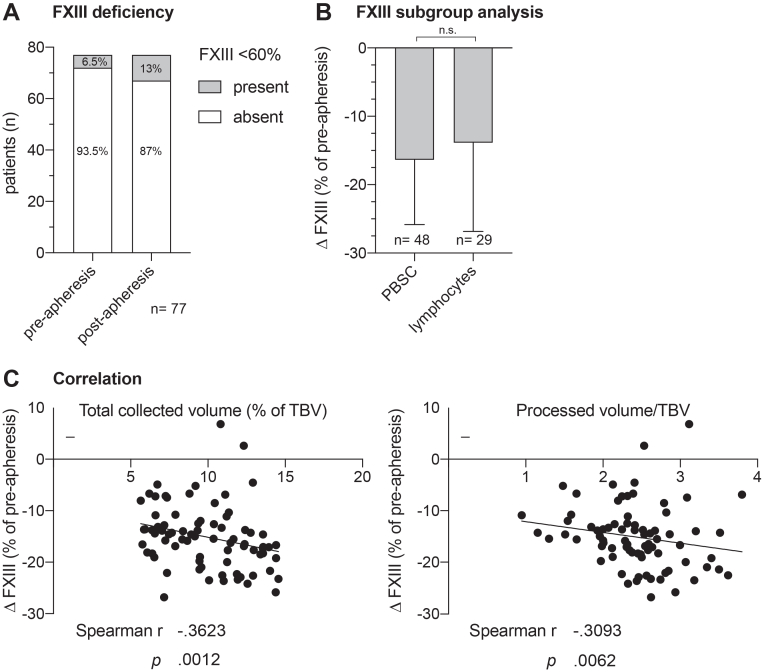
FXIII deficiency is common in patients undergoing cell apheresis. All panels: Level of significance .05. (A) Percentage of patients (*n* = 77) with factor XIII (FXIII) deficiency (defined as FXIII activity <60%) pre- and postapheresis. *P* value from Fisher’s exact test was 0.2768. (B) Procedure-related FXIII loss, defined as the percentage of preapheresis FXIII activity, in patients undergoing apheresis for peripheral blood stem cells (PBSCs) (*n* = 48) and lymphocytes (*n* = 29). *P* value from Mann–Whitney test was 0.1719. (C) Left panel: Correlation of FXIII loss with total collected volume including plasma (calculated as percentage of total blood volume [TBV]). Right panel: Correlation of FXIII loss with the ratio of processed volume and TBV. The strength of the association between FXIII loss (defined as the percentage of preapheresis FXIII activity) and procedural data was calculated by Spearman rank correlation, and the functional relationship was described by linear regression analysis, as shown in the figure.

Before apheresis, 5 of 77 patients (6.5 %) were FXIII deficient (defined as FXIII activity <60%). These were 2 female patients with lymphoma undergoing lymphocyte apheresis, 2 male patients with multiple myeloma undergoing PBSC apheresis, and 1 male patient with Ewing’s sarcoma undergoing PBSC apheresis. After apheresis, 10 patients (13.0 %) met the criteria for FXIII deficiency (*P* = .28; Figure 3A). FXIII loss, defined as the percentage of postapheresis FXIII activity relative to preapheresis levels, was comparable between the PBSC and lymphocyte apheresis cohorts (*P* = .17; Figure 3B), under the limitation that the study was not specifically designed for subgroup analysis. We next examined the correlation between FXIII loss, defined as the percentage decrease in postapheresis FXIII activity relative to preapheresis levels, and procedural data. We observed an inverse correlation of FXIII loss with collected volume (percentage of TBV; Spearman *r* = −0.3623, *P* = .001) and with processed volume/TBV (Spearman *r* = −0.3093, *P* = .006; Figure 3C). However, no correlation was found with total collected volume (Spearman *r* = −0.1869, *P* = .10), processed volume (Spearman *r* = −0.04602, *P* = .69), infused ACD-A (Spearman *r* = −0.04095, *P* = .72), apheresis time (Spearman *r* = −0.1406, *P* = .22), EFB (Spearman *r* = −0.03417, *P* = .77), and EFB/TBV(%) (Spearman *r* = −0.07040, *P* = .54; Supplementary Figure 3).

### 3.7 Adverse events

One patient presented with an abscess in the upper back region due to a superinfected atheroma during stem cell mobilization, requiring a surgical incision the day before stem cell collection. After apheresis, bleeding from the wound occurred when the tamponade was removed. Another surgical procedure revealed bleeding from a venous stump that was successfully cauterized. Subsequent coagulation factor analysis showed reduced FXIII activity of 47% before apheresis, which was further reduced to 43% after apheresis. In addition, the patient presented with thrombocytopenia (platelet count 58 × 10^9^/L preapheresis, 51 × 10^9^/L postapheresis). All other analyzed coagulation parameters were normal. FXIII (1250 IU) was administered and 1 platelet concentrate was transfused. Broad-spectrum antibiotics were initiated. The next day, FXIII activity increased to 67% and platelet count rose to 83 × 10^9^/L. The patient made a rapid and sustained recovery.

### 3.8 Process-related dilution

Citrate plasma from 3 healthy volunteer donors was diluted with ACD-A buffer and saline at 5% and 10%, respectively, and analyzed for global parameters (PT and PTT), fibrinogen concentration, VWF concentration, and FXIII and VWF activities. In contrast to our previous observations before and after apheresis, activities and concentrations decreased linearly with increased dilution, in both single parameters and global tests. The results are presented in the Supplementary Table and, for FXIII, in Supplementary Figure 5. No relevant differences in activities and concentrations were observed upon dilution with saline or ACD-A.

## 4 Discussion

We present a prospective and comprehensive analysis of how preparative apheresis, beyond cellular components, affects coagulation factors and platelet function.

While global parameters of coagulation (PTT and INR) remained largely unchanged and within reference ranges, we observed a significant loss of the individual components fibrinogen, FXIII, antithrombin, VWF antigen, and VWF activity after apheresis. These results are consistent with previous work showing that stem cell collection from healthy allogeneic donors was associated with a prolongation of PT, whereas PTT and TT were unaffected [6]. Of particular interest in the present study is the observed loss of VWF antigen and activity. Since VWF is an acute phase protein [19] and G-CSF-dependent upregulation has been demonstrated in allogeneic donors [20], our results suggest that either the apheresis procedure does not induce VWF levels per se or that the loss associated with the procedure outweighs the upregulation of VWF in the acute phase. Through their analysis of rotational thromboelastometry, Cilek et al. [21] discovered that stem cell collection temporarily affects hemostatic function, likely due to a reduction in coagulation factors and a loss of platelets. Of note, no sustained hypercoagulable state was observed after apheresis [21].

The primary focus of the study was FXIII, given its critical role in clot stabilization [[22], [23], [24], [25]], the fact that FXIII it is not covered by global coagulation tests [17], and that FXIII deficiency has been reported in several diseases, including in patients with multiple myeloma [16]. A retrospective study found that patients undergoing therapeutic plasma exchange developed FXIII deficiency and hemorrhages [26]. Bleeding events have been recorded, particularly in patients with a history of surgery [26,27]. So far, FXIII has not been evaluated in the context of cell apheresis. In the absence of data, our primary hypothesis was that FXIII activity would decrease by 20% after apheresis. This hypothesis was not confirmed; however, we did find a 15% reduction in FXIII activity. The decrease in FXIII activity correlated with both the TBV-related processed volume and the TBV-related collected volume, indicating that patient-related factors must be considered when estimating the loss of plasmatic components. We did not find a correlation with the EFB, which suggests that dilution may not be the main cause of the results.

FXIII is a Ca^2+^-dependent transglutaminase with 3 highly conserved Ca^2+^-binding sites, whose coordinated occupancy is crucial for conformational change and substrate binding [28]. In most of our patients, calcium levels were only moderately reduced after apheresis and generally sufficient for physiological coagulation. Despite the common presumption that laboratory parameters of coagulation should be independent from at least slight changes in patients’ calcium levels, it cannot completely be excluded that ACD-A-induced decreases in calcium concentration postapheresis could potentially have affected the measurements. In this case, the observed decrease in coagulation factors after apheresis might be overestimated in relation to the actual physiological change. However, there is only limited published data on the effect of ACD-A on coagulation parameters [29], an underexplored field that requires further investigation. Yamada et al. [30] demonstrated a transient citrate effect on routine coagulation tests after therapeutic plasma exchange. However, the procedures are not entirely comparable. For our study, this aspect seems irrelevant for the quantification of VWF antigen and activity due to turbidimetric measurements and similarly for antithrombin due to the strong dilution of the original plasma in the reaction mixture.

The dilution of citrate plasma *in vitro* with ACD-A and saline, respectively, affected single coagulation factors and global parameters in a linear fashion. In particular, we saw no evidence of a citrate-dependent reduction in FXIII activity. However, the global coagulation parameters remained unaffected *in vivo* after apheresis, maybe as an expression of quick rebalancing of the overall coagulation system in spite of a protracted reduction in single factor activities.

The largely similar decrease in individual components determined by different methods strongly suggests that the observed effect is mainly due to plasma loss during apheresis.

The detected loss of the endogenous anticoagulant antithrombin, which occurs to a comparable extent, implies that this observation can be extended to other coagulation factors not analyzed in our study. Nevertheless, possible effects of the observed reduction of antithrombin on global hemostasis remain speculative. In patients with reduced activities of specific coagulation factors such as FX in patients with amyloidosis, the additional loss—and the estimated amount to be substituted—can be assessed by the use of data such as those presented in our study.

In terms of cellular components, a relevant proportion of platelets was removed during leukapheresis, an observation consistent with previous studies showing 20% to 40% reduction in platelets after PBSC collection [2,4,31], mainly due to the close proximity of the platelet layer to the mononuclear layer [2,32]. In contrast to the platelet count, only a small loss of hemoglobin was observed after apheresis.

When platelet function was assessed by impedance aggregometry (Multiplate assay), reduced platelet aggregation was observed after stimulation with arachidonic acid, ADP, and TRAP in the entire cohort. This was also the case when only patients with platelet counts >100 × 10^9^/L were considered, demonstrating a consistent and nonspecific reduction in platelet function after apheresis. The reasons for this observation warrant further research. A prospective study demonstrated that extracorporeal venovenous membrane oxygenation initially leads to reduced platelet aggregation and activation, with recovery occurring at least partially over time [33,34]. Furthermore, apheresis-derived platelet concentrates show less evidence of platelet activation during storage than buffy coat-derived concentrates [35], although differences can be found in specific apheresis platforms [36].

Regarding adverse events, only 1 minor bleeding event was observed in our study, which illustrates the overall safety of the apheresis procedure and is consistent with previous work [37]. In this case, the bleeding occurred after apheresis following surgery, and the only laboratory finding was a reduced FXIII activity before and after apheresis. The patient underwent a surgical revision to stop the bleeding and received 1250 units of FXIII, which normalized FXIII activity. Yet, it remains elusive to which extent the FXIII deficiency observed here contributed to the bleeding complication.

In conclusion, we show that a more detailed analysis is able to reveal distinct changes in hemostasis and platelet function related to cell apheresis, which remain undetected by global coagulation parameters. As a rule of thumb, a reduction in coagulation factor activity and/or concentration of approximately 15% to 20% should be expected. When a factor deficiency is known or bleeding occurs, the observed changes in coagulation factor activity as well as in platelet function deserve increased attention.
