# Supplementary material for: Preparative leukapheresis impairs autologous donor hemostasis and platelet function
Source: Res Pract Thromb Haemost. 2026 Jul 13;10(5):106819. doi: 10.1016/j.rpth.2026.106819 (PMC13474639; doi:10.1016/j.rpth.2026.106819)
Supplement: Supplementary file 1 [file mmc1.pdf]

Preparative leukapheresis impairs autologous donor haemostasis and platelet function

Veronika Dill[1,2], Philipp Blüm[1,3], Laura-Vanessa Stegmaier Diaz[1], Georg Mößmer[4], Markus Thaler[4], Patrick Möhnle[5], Florian Bassermann[1,2,6,7] and Martin Hildebrandt[1,7]

[1]TUM School of Medicine and Health, TUM University Hospital, Klinikum rechts der Isar, Department of Internal Medicine III, Technical University of Munich, Munich, Germany
[2]German Cancer Consortium (DKTK), partner site Munich, Munich, Germany
[3]Institute of Transfusion Medicine and Immunology, Medical Faculty Mannheim, Heidelberg University, German Red Cross Blood Service Baden-Württemberg-Hessen, Mannheim, Germany
[4]TUM School of Medicine and Health, TUM University Hospital, Klinikum rechts der Isar, Institute of Clinical Chemistry and Pathobiochemistry, Technical University of Munich, Munich, Germany
[5]Division of Transfusion Medicine, Cell Therapeutics and Hemostaseology, University Hospital, Ludwig Maximilian University, Munich, Germany
[6]Center for Translational Cancer Research (TranslaTUM), School of Medicine, Technical University of Munich, Munich, Germany
[7]Bavarian Cancer Research Center (BZKF)

Corresponding author: Martin Hildebrandt, MD
TUM University Hospital
Klinikum rechts der Isar
Technical University of Munich
Department of Internal Medicine III
Ismaninger Straße 22
81675 Munich, Germany
Tel: +49-89-4140 4111
Email: martin.hildebrandt@mri.tum.de

Supplementary Figure 1

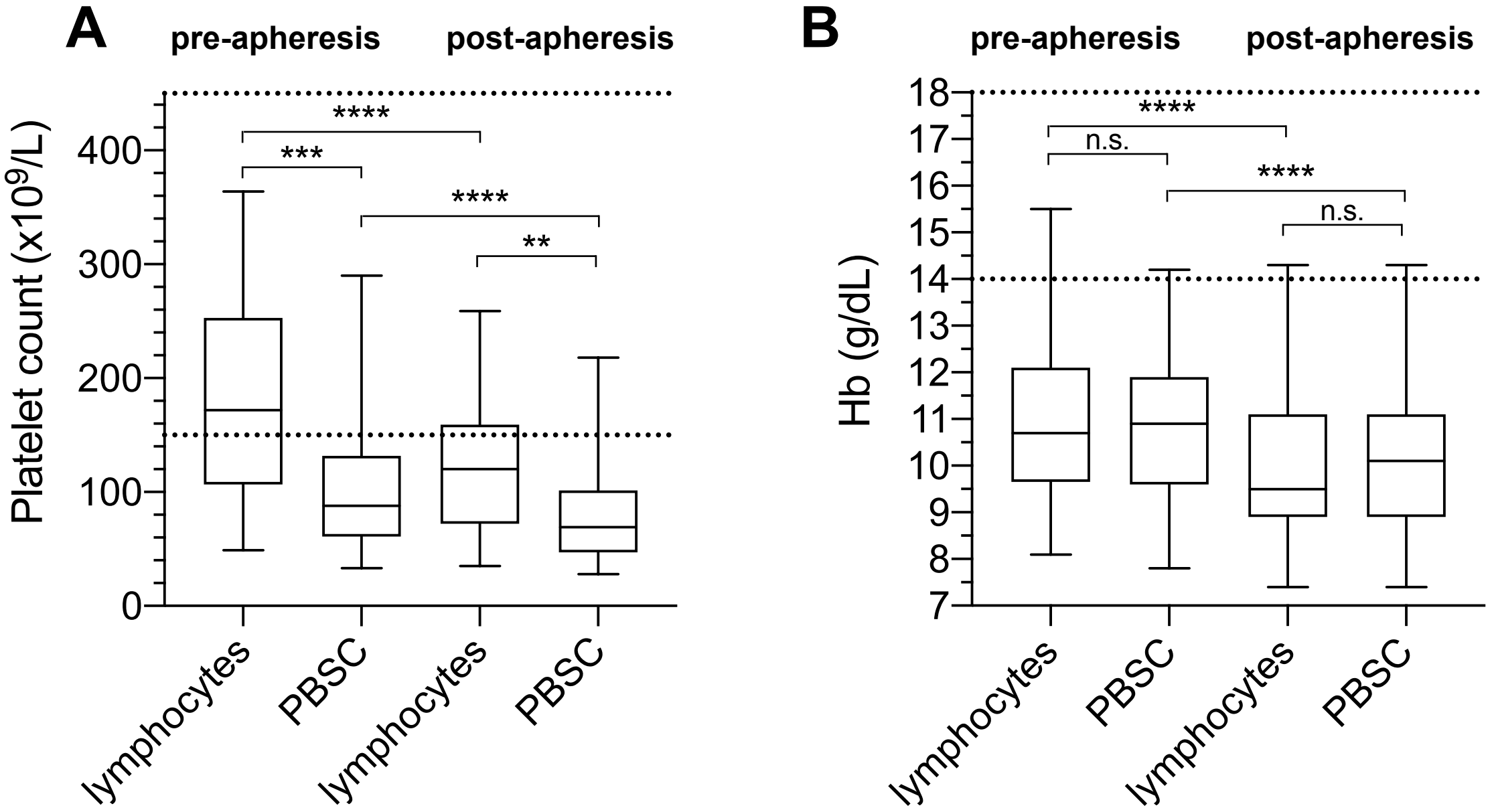

Changes in platelet counts and haemoglobin levels in patients undergoing PBSC apheresis and lymphocyte apheresis. All panels: Box plots show median and interquartile range (25th and 75th). Whiskers indicate minimum and maximum values analysed. The respective reference ranges of the Institute of Clinical Chemistry and Pathobiochemistry are shown as dotted lines. Only patients with both pre- and post-apheresis values available were included. The pre- and post-apheresis values were analysed by paired t-test. Level of significance .05, ** *p* <.0.01, *** *p* <.001, **** *p* <.0001. Welch`s t test was used to compare the PBSC and lymphocyte cohorts. (A) Changes in platelet count from pre- to post-apheresis in the lymphocyte cohort (n=29) and in the PBSC cohort (n=48). Lymphocytes pre-apheresis vs. lymphocytes post-apheresis *p* <.0001. PBSC pre-apheresis vs. PBSC post-apheresis *p* <.0001. Lymphocytes pre-apheresis vs. PBSC pre-apheresis *p* =.0004. Lymphocytes post-apheresis vs. PBSC post-apheresis: *p* =.0041. (B) Changes in haemoglobin from pre- to post-apheresis in the lymphocyte cohort (n= 29) and in the PBSC cohort (n=51). Lymphocytes pre-apheresis vs. lymphocytes post-apheresis *p* <.0001. Lymphocytes pre-apheresis vs. PBSC pre-apheresis *p* =.9383. PBSC pre-apheresis vs. PBSC post-apheresis *p* <.0001. Lymphocytes post-apheresis vs. PBSC post-apheresis *p* =.3889. PBSC: Peripheral blood stem cells.

Supplementary Figure 2

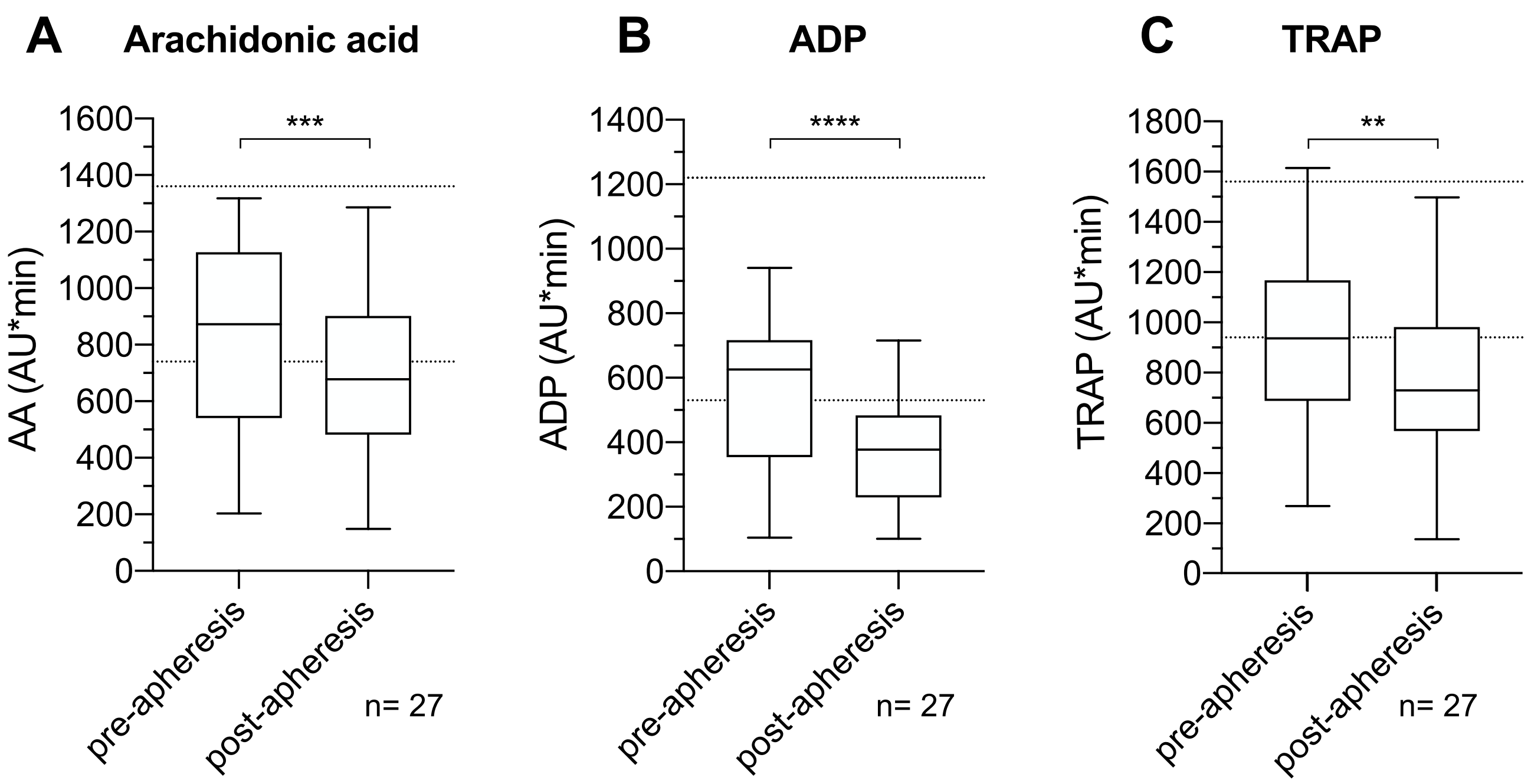

Platelet aggregometry in patients with platelet counts > 100 x 10^9/L. All panels: Only patients with platelet counts > 100 x 10^9 G/L both before and after apheresis were included. Box plots show the median and interquartile range (25th and 75th). Whiskers indicate minimum and maximum values analysed. The individual reference ranges of the Institute of Clinical Chemistry and Pathobiochemistry are shown as dotted lines. The number of samples analysed is shown in the figure. Pre- and post-apheresis values were analysed by paired t-test. Level of significance .05, ** p <.0.01, *** p <.001, **** p <.0001. Changes in platelet aggregation as determined by the area under the curve (AU*min) of impedance aggregometry (Multiplate®) after stimulation with (A) arachidonic acid (p =.0003), (B) adenosine diphosphate (ADP) (p <.0001), and (C) thrombin-activated peptide-6 (TRAP) (p = .0014) from pre- to post-apheresis.

Supplementary Figure 3

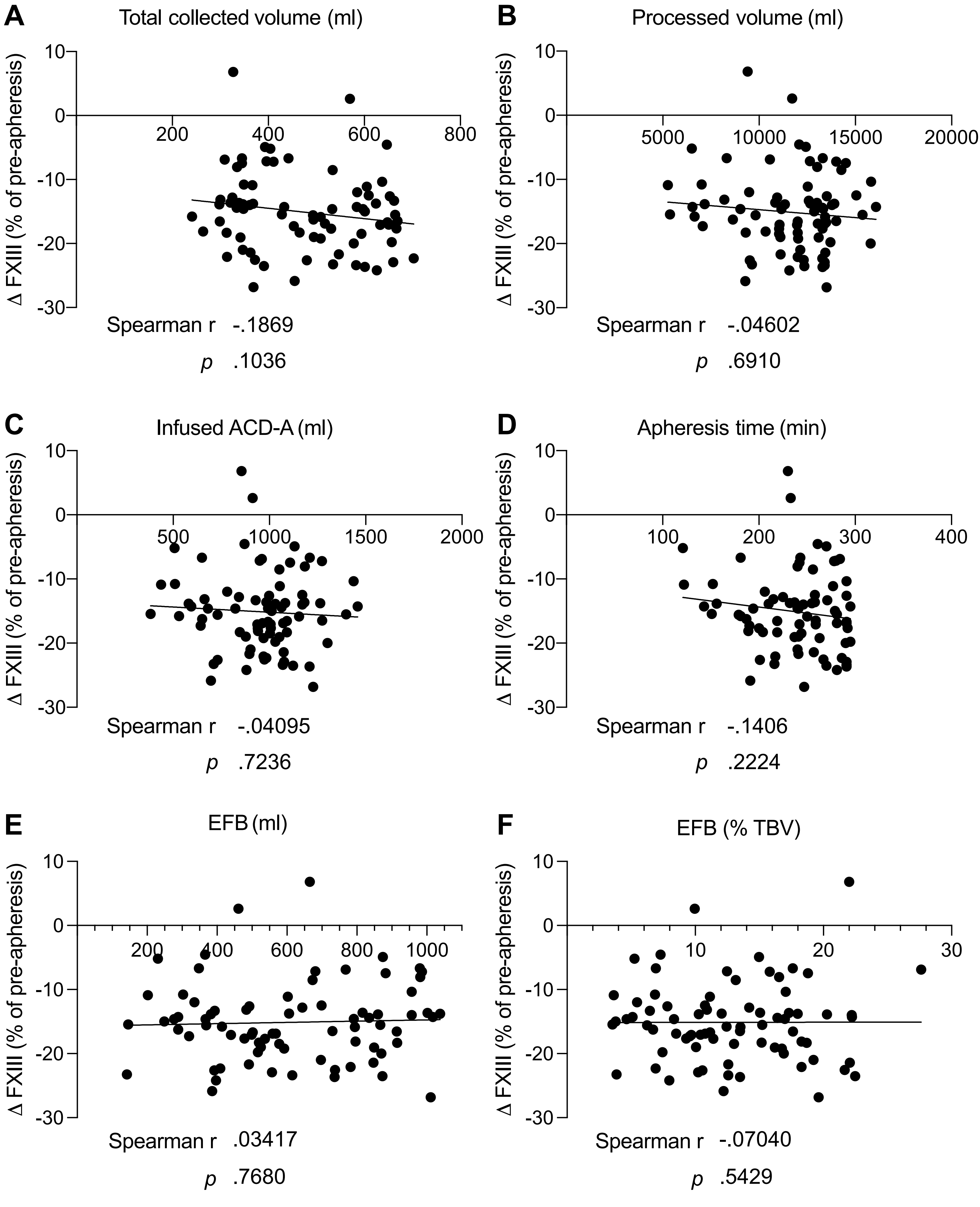

Correlation of FXIII loss with procedural data. All panels: The strength of the association between FXIII loss (defined as the percentage of pre-apheresis FXIII activity) and procedural data was calculated by Spearman rank correlation, and the functional relationship was described by linear regression analysis, as shown in the figure. Level of significance .05. (A) Correlation of FXIII loss with total collected volume including plasma (ml). (B) Correlation of FXIII loss with processed volume during apheresis (ml). (C) Correlation of FXIII loss with infused acid citrate dextrose formula-A (ACD-A) (ml) during apheresis. (D) Correlation of FXIII loss with apheresis time (min). (E) Correlation of FXIII loss with EFB (ml). (E) Correlation of FXIII loss with EFB (% of TBV). EFB: End fluid balance. FXIII: factor XIII. TBV: Total body volume

Supplementary Figure 4

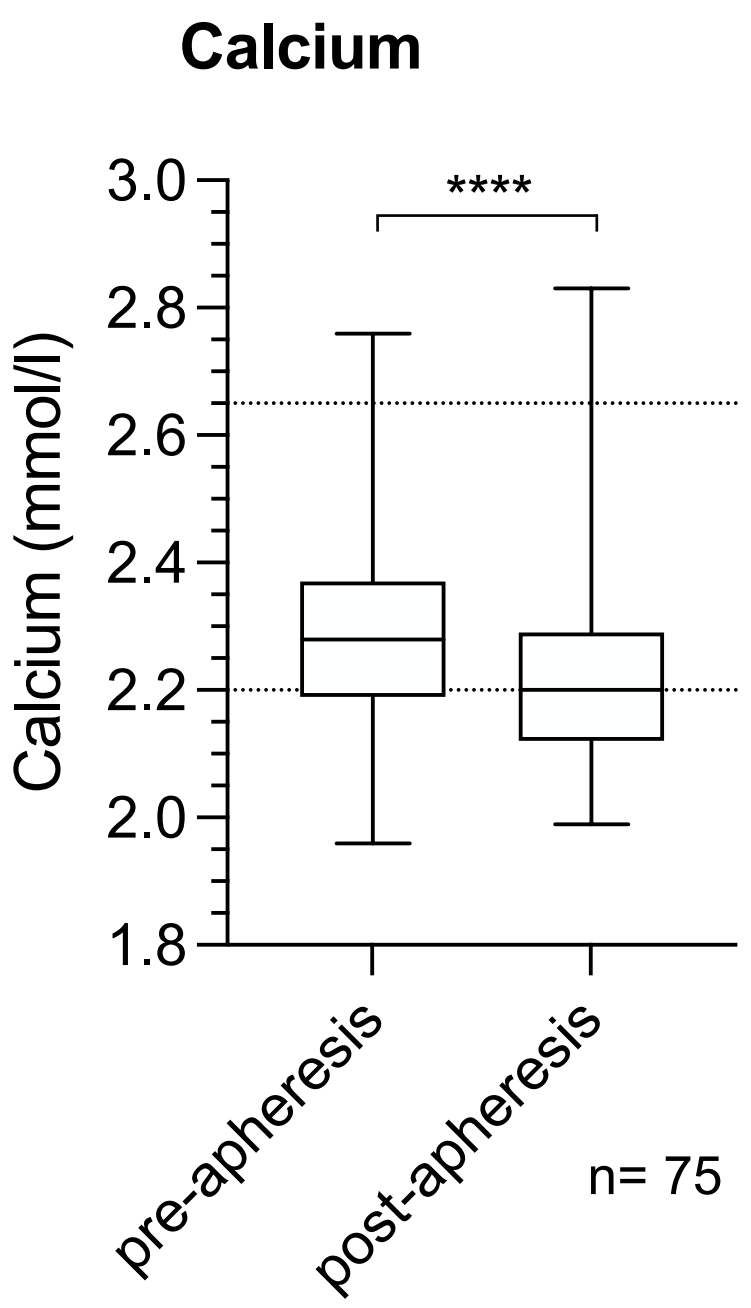

Changes in serum calcium levels after apheresis. Box plots show the median and interquartile range (25th and 75th). Whiskers indicate minimum and maximum values analysed. The reference range of the Institute of Clinical Chemistry and Pathobiochemistry is shown as dotted lines. Only patients with both pre- and post-apheresis values available were included. The number of samples analysed is shown in the figure. Pre- and post-apheresis values were analysed by paired t-test. Level of significance .05, **** p <.0001.

Supplementary Figure 5

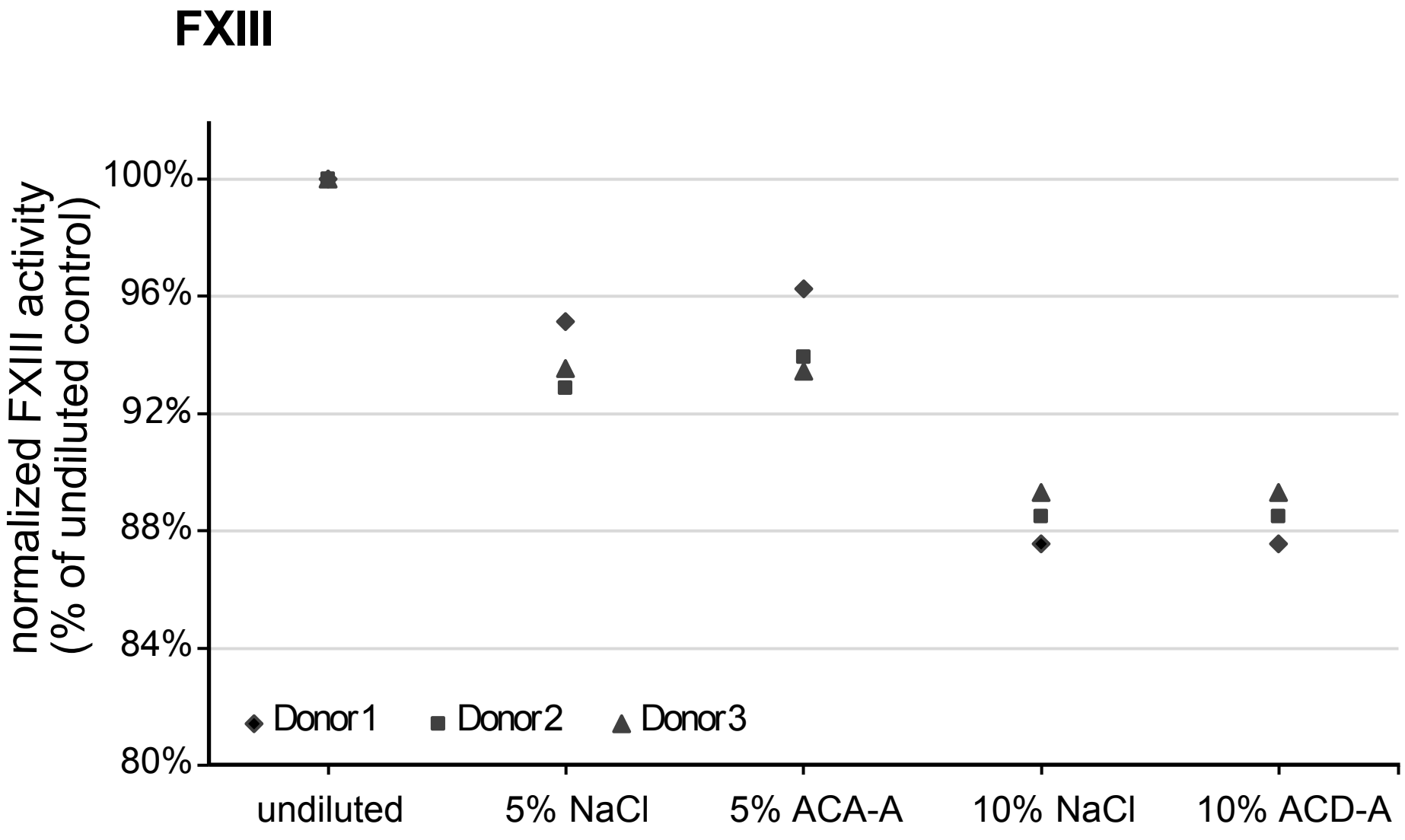

Normalized FXIII activity across dilution conditions. For each healthy volunteer donor, the activity of FXIII was determined in duplicate using 5% and 10% ACD-A buffer and saline solution, respectively. The mean of these values was normalized to the mean of the undiluted condition. The dots represent the mean normalized FXIII activity of each donor. FXIII: Factor XIII. NaCl: Saline solution.

Supplementary Table

	Donor 1					Donor 2					Donor 3				
	undiluted	5% NaCl	5% ACD-A	10% NaCl	10% ACD-A	undiluted	5% NaCl	5% ACD-A	10% NaCl	10% ACD-A	undiluted	5% NaCl	5% ACD-A	10% NaCl	10% ACD-A
PT [%] (1)	117,4	111,3	109,9	108,1	86,6	97,6	95,7	87,3	91,3	60,1	112,8	108,3	112,0	104,1	95,2
PT [%] (2)	117,0	111,1	112,1	107,5	90,0	96,3	93,9	88,4	90,1	64,8	113,7	107,7	109,4	103,0	90,9
PT [%] MV	117,2	111,2	111,0	107,8	88,3	97,0	94,8	87,9	90,7	62,5	113,3	108,0	110,7	103,6	93,1
INR (1)	0,90	0,94	0,95	0,96	1,10	1,02	1,04	1,09	1,07	1,33	0,93	0,96	0,93	0,98	1,04
INR (2)	0,90	0,94	0,93	0,96	1,07	1,03	1,05	1,08	1,07	1,28	0,92	0,96	0,95	0,99	1,07
INR MV	0,90	0,94	0,94	0,96	1,09	1,03	1,05	1,09	1,07	1,31	0,93	0,96	0,94	0,99	1,06
PTT [s] (1)	29,1	30,1	30,4	30,4	34,0	29,8	30,7	31,8	31,2	35,5	29,0	28,9	30,3	29,0	33,1
PTT [s] (2)	29,1	29,6	32,1	30,6	33,5	30,3	30,8	32,2	30,3	36,1	29,1	29,0	30,2	29,1	33,2
PTT [s] MV	29,1	29,9	31,3	30,5	33,8	30,1	30,8	32,0	30,8	35,8	29,1	29,0	30,3	29,1	33,2
FIB [mg/dl] (1)	292	281	272	267	252	236	233	226	224	215	367	358	342	339	312
FIB [mg/dl] (2)	301	284	287	271	251	237	231	230	220	213	352	347	336	327	299
FIB [mg/dl] MV	297	283	280	269	252	237	232	228	222	214	360	353	339	333	306
FXIII [%] (1)	80,3	74,4	75,9	67,9	69,2	109,1	101,1	101,3	95,9	96,4	143,8	136,2	134,3	128,8	124,6
FXIII [%] (2)	79,5	77,6	77,9	71,8	70,7	108,1	100,6	102,7	96,0	95,8	144,0	133,0	134,6	124,0	132,4
FXIII [%] MV	79,9	76,0	76,9	69,9	70,0	108,6	100,9	102,0	96,0	96,1	143,9	134,6	134,5	126,4	128,5
vWF Act. [%] (1)	70,1	62,9	66,4	63,4	62,2	150,0	147,8	142,9	139,8	133,0	125,6	123,2	125,2	113,9	112,9
vWF Act. [%] (2)	71,0	67,1	66,8	64,9	63,2	145,4	145,3	147,1	138,3	145,1	123,6	126,2	122,6	116,3	111,3
vWF Act. [%] MV	70,6	65,0	66,6	64,2	62,7	147,7	146,6	145,0	139,1	139,1	124,6	124,7	123,9	115,1	112,1
vWF Ag [%] (1)	75,7	72,4	71,6	68,6	69,0	158,3	147,7	150,0	138,2	140,6	111,6	103,6	102,7	98,7	99,4
vWF Ag [%] (2)	79,3	70,8	73,0	68,5	70,0	156,1	144,3	147,3	137,2	138,2	109,7	101,9	103,5	96,3	97,4
vWF Ag [%] MV	77,5	71,6	72,3	68,6	69,5	157,2	146,0	148,7	137,7	139,4	110,7	102,8	103,1	97,5	98,4

Results of dilution measurements using saline and ACD-A in three healthy volunteer donors. Measurements were performed in duplicates at the indicated concentrations, and individual values as well as mean values (MV) are shown. FIB: Fibrinogen. FXIII: Factor XIII. INR: International normalized ratio. NaCl: Saline solution. PT: Prothrombin time. PTT: Partial thromboplastin time. vWF Act: von Willebrand factor activity. vWF Ag: von Willebrand factor antigen.
